# Mobile Synchronization Recovery for Ultrasonic Indoor Positioning

**DOI:** 10.3390/s20030702

**Published:** 2020-01-27

**Authors:** Riccardo Carotenuto, Massimo Merenda, Demetrio Iero, Francesco G. Della Corte

**Affiliations:** 1 DIIES Department, University Mediterranea of Reggio Calabria, 89126 Reggio Calabria, Italy; r.carotenuto@unirc.it (R.C.); demetrio.iero@unirc.it (D.I.); francesco.dellacorte@unirc.it (F.G.D.C.); 2 HWA srl, Spin-off University Mediterranea of Reggio Calabria, Via R. Campi II tr. 135, 89126 Reggio Calabria, Italy

**Keywords:** synchronization recovery, TOA, TDOA, mobile device positioning, 3D ultrasonic positioning

## Abstract

The growing interest for indoor position-based applications and services, as well as ubiquitous computing and location aware information, have led to increasing efforts toward the development of positioning techniques. Many applications require accurate positioning or tracking of people and assets inside buildings, and some market sectors are waiting for such technologies for starting a fast growth. Ultrasonic systems have already been shown to possess the desired positioning accuracy and refresh rate. However, they still require accurate synchronization between ultrasound emitters and receivers to work properly. Usually, synchronization is carried out through radio frequency (RF) signals, adding system complexity and raising the cost. In this work, this limit is overcome by introducing a novel self-synchronizing indoor positioning technique. Ultrasonic signals travel from emitters placed at fixed reference positions to any number of mobile devices (MD). The travelled distance is computed from the time of flight (TOF), which requires in turn synchronism between emitter and receiver. It is shown that this synchronism can be indirectly estimated from the time difference of arrival (TDOA) of the ultrasonic signals. The obtained positioning information is private, in the sense that the positioning infrastructure is not aware of the number or identity of the MDs that use it. Computer simulations and experimental results obtained in a typical office room are provided.

## 1. Introduction

Accurate 3D real-time positioning with high spatial and temporal resolution is pursued by a growing number of research projects and industry teams. A number of ready for growth markets are waiting for reliable, fast, and affordable technologies capable of providing tridimensional coordinates for people and objects indoors. This large audience is looking for the indoor equivalent of what is the Global Positioning System (GPS) for open air spaces, but with better tracking accuracy and higher refresh rate. In fact, positioning from satellite services such as GPS or Galileo is inadequate inside closed environments due to the lack of line-of-sight and low signal level, while also accuracy and refresh rates are insufficient. Applications include man-machine gestural interfaces, marketing, and navigation in closed environments such as malls, hospitals, airports, etc., virtual and augmented reality for gaming, museum and historical heritage sites, domotics by controlling home appliances with human presence and movements, security by controlling access, tracking persons, and monitoring assets, etc. [1,2,3,4,5,6,7,8]. It is worth noting that many of these applications require, in addition to absolute positioning, also the trajectory of motion with sufficient repeatability and smoothness to derive high quality gestural features or vectors, rather than high absolute positioning accuracy.

To date, no indoor positioning technology well suited for all purposes has emerged as a final winning solution, and the research field is still fully open. Many attempts to develop indoor positioning systems have been reported in the literature [9], but none of them seems sufficiently accurate, low cost, miniaturizable, and showing at the same time energy consumption compatible with small batteries. Proposed systems rely on a number of technologies including Radio Frequency (RF) signals [10], multiple cameras, lasers [11], infrared beams [12], magnetic sensors [13], and ultrasonic signals [14]. Among these techniques, systems based on ultrasonic waves can provide distances and spatial positions with a high degree of precision at a relatively low cost [15].

The most used techniques are time of arrival (TOA) and time difference of arrival (TDOA). TOA based methods require knowing exactly when (time of emission, TOE) the emitter starts to emit the ultrasonic signal, and thus require a tight synchronization between emitter and receiver, usually obtained using a suitable RF communication channel. In such systems, ultrasonic and reference Radio Frequency (RF) signals are emitted synchronously at a given TOE, and distances are estimated by neglecting the propagation time of the RF signal, so that the TOF = TOA- TOE is the time elapsed from the RF emission at TOE to the reception TOA of the ultrasonic signal with an excellent degree of approximation. This implies the knowledge of both TOE and TOA at the place where the TOF is estimated. Often TOE is transmitted through a trigger signal from the emitter to the MD using a RF or infrared (IR) additional channel. The emitter-receiver distance is then computed by multiplying this TOF and the known propagation speed of sound in air [16,17]. The comparatively low propagation velocity of ultrasonic waves in air allows high accuracy of signal’s time of flight (TOF) measurement, and thus high accuracy ranging and positioning. TOF ultrasonic positioning systems use true range multilateration for the spatial positioning of a mobile device (MD) starting from the measurements of the distance between the MD and a set of reference points. Unfortunately, in such positioning systems, the inclusion of an RF or IR subsystem to provide a time trigger signal adds significant complexity, size, power consumption, and cost [18].

Knowing the distances between MD and reference points (at least three for 3D positioning in a hemispace) allows finding the position of the MD as the intersection of spheres centered at the reference points, which implies the solution of a set of nonlinear equations. There are many approaches to solving these nonlinear equations: Direct geometric solution, minimization of a suitable cost function, or statistical methods (Bayesan methods, Kalman filtering) [19]. Geometric methods are based on the linearization of the system of equations in order to find a mathematically closed form solution [20,21]. When it is allowed to appropriately choose the number and arrangement of the emitters, the system of nonlinear equations can be solved directly [22]. The main problem with these methods is noise sensitivity in some positioning areas.

In the absence of any synchronization between emitter and receiver, it is possible to obtain a positioning with pseudo range multilateration by only measuring TOAs. The technique is based on the differences in the TOAs of two or more received signals, using one of them as a reference, thus obtaining TDOAs. Successively, the TDOAs are converted in differences of distances. At least three distance differences from four reference points are needed to obtain the tridimensional MD positioning as an intersection of three of hyperboloids. In [23], a solution based on both TDOA and frequency difference of arrival (FDOA) for including target speed information was presented. The hyperbolic multilateration problem can also be approached using the iterative numerical approach based on Cayley–Menger bideterminant [24], but with a significant computational cost, especially considering small and battery-operated sensors. Moreover, for numerical iterative methods, it is essential for convergence, but not always viable, to provide a good initial guess.

In [25], the experimental 2D positioning for indoor navigation of smartphones acting as acoustical receivers is reported. TDOA positioning is achieved using an iterative least square matrix method. Sub-decimeter positioning accuracy was reported together with an analysis of the acoustic reception quality related to the rotation angle of the smartphone in respect to the emitters.

In [26], many beacons were placed in unknown positions, and statistical and iterative methods, through linear approximations of highly nonlinear equations (GN, LM, particle filter, Kalman filter etc.) were employed to obtain the position of the receiver. 2D positioning accuracy in the order of tens of centimeters was reported.

In [27], a simultaneous 2D TDOA positioning of many transmitters and a moving receiver is presented. Positioning is obtained iteratively solving a nonlinear optimization problem of TDOA using a physical spring–mass representation of the error function. However, this solution shows a high computational cost and a consequent large footprint for the powerful processor required and its large battery.

In [28], an algebraic solution of the 2D TDOA positioning is provided using more than four reference points. It is also shown that in regions of poor geometric dilution of precision (GDOP), the algebraic solution always performs better than an iterative solution derived from linear approximation of a nonlinear computation. In [29] a mathematically closed form is derived for TDOA positioning, analyzing ambiguity issues of the set of possible solutions. 

In [30], three-dimensional positioning is obtained using five reference points or beacons and thus four TDOAs. This redundancy allows equation linearization and therefore algebraic solution. Simulative results were also reported therein. In [31], a room level positioning system for mobile robots navigation was reported, with experimental results in the floor plane (2D positioning). In [32], a low cost ultrasonic based positioning system for indoor mobile robots navigation was described. This system was intended for distribution at floor level and was based on a least squares (LS) method for calculating positioning. TOAs were obtained with dynamic threshold detection, and operation was partially limited by the narrow emission cone of the employed transducers. A positioning dilution of precision (PDOP) analysis depending on the relative position between MD and reference points was also provided.

A recently proposed indoor ultrasonic positioning system uses a sort of TOF positioning without using reference signals of any kind to signal the ultrasonic TOE [33]. It employs a hybrid combination of angle of arrival (AOA) and TOF with a clever timing recovery algorithm that is able to indirectly estimate the correct timing during operation. Recovery of synchronization is obtained in two steps. An array of at least three receivers allows to estimate the direction of origin of the signal from a given ultrasonic beacon, placed in a reference point. With three beacons transmitting at known and fixed rates, the three estimated directions through an angulation method produce a coarse estimate of the 3D position of the MD. The coarse MD-beacon distances and the estimated times of arrival (TOA) allow to estimate the TOEs of the ultrasonic signals. Since the emission timing sequences are known, the estimated TOEs determine the timing offset (TO) estimates of the beacon emission sequences, which are constant relative to the MD’s clock. As a consequence, the TO estimates can be averaged over a number of ultrasonic emission periods. Assuming that the TO estimates relative to the MD’s clock are noisy with their mean equal to the true TO, a moving average will converge over time to the true TO. Based on this, a more accurate estimate of the MD location is then obtained through a conventional TOF method, where the TOF is computed using the measured TOAs and the TOEs estimates from the converged TO. Ultimately, the averaging of the TO estimates ensures a reduced estimation noise, in turn allowing for a more accurate estimate of the position based on TOF, e.g., using the intersection of spheres.

Despite the clever design, this system still presents some critical issues. In fact, in real systems, the beacon clock and the MD clock, although nominally at the same frequency, show a relative clock drift, due among other things to manufacturing tolerances, temperature, and aging. The clock drift causes an event that happens at the beacons at constant intervals to be seen by the MD as happening at slowly increasing or decreasing time intervals over time. Therefore, over time the TO is not seen by the MD as a constant, but it is seen as a time ramp. For this reason, the estimate of TO computed with a simple moving average does not converge to the true value. Furthermore, the need to equip the MD with at least three ultrasonic receivers does not favor a compact and low-power design. Moreover, the reported iterative computation load for positioning is not easily achievable with a low power design.

In this work, a system is proposed that, inspired by that described above [33], overcomes some of its critical elements, namely the use of three receiving microphones and the clock drift. Our system uses four ultrasonic beacons and the MD has only one ultrasonic receiver. Periodically, the four beacons emit a sequence of ultrasonic signals, regardless of the presence or absence of MDs. All the positioning computations are performed onboard each MD.

In a first step, a moving MD estimates the TO using only TDOA information using a mathematically closed form, in this work derived from the intersection of hyperboloids, which however is notoriously highly sensitive to measurements’ uncertainty [34,35,36]. The MD estimates TO from scratch, i.e., without prior information. As explained, due to the unavoidable clock drift, the MD does not see a constant TO with respect to its clock, but a time ramp with slope proportional to the clock drift. It is therefore not possible to obtain a convergent estimate of true TO using simple moving averages. Hereafter, a new solution of the above problem using a suitable ramp follower that converges even in the presence of clock drift is also presented.

In the second step, a more accurate estimate of the MD position is obtained through spheres’ intersection, whose radii are obtained through converged TO estimates and measured TOAs. Both TO estimation and spherical positioning calculations are performed using two closed form formulas, thus minimizing the computational load with significant savings on complexity, size, and energy consumption of the MD.

The main advantage of this method is the elimination of the trigger signal between beacons and MD, and consequently of the one-way RF communication from the beacon set to the MD. The elimination of the RF section leads to a reduction in the complexity, size, and power consumption of both the beacon set and the MD. Furthermore, the proposed method does not present convergence problems, has a much lower computational complexity than iterative or statistical methods and a lower sensitivity to noise compared to other geometric methods based on linearization (see [19,20,21] and [23,24,25,26,27,28,29,30,31,32]).

Similarly to the GPS system for open-air environment, this system can be used in an indoor environment by a variety of different devices that meet a pre-established standard. So, in perspective, special purpose MD, smartphones, tablets, notebooks could exploit the existence of a common indoor positioning structure running a simple software application and easily getting indoor positioning and navigation services in certain places such as malls, hospitals, and airports.

This paper is structured as follows. Section 2 explains the proposed system and algorithm. Section 3 reports the preliminary simulations and the experimental results. Section 4 draws the paper conclusions.

## 2. System Architecture and Synchronization Recovery

In this section, an innovative solution to the MD timer synchronization with the Beacons emission is presented.

**Figure 1:**
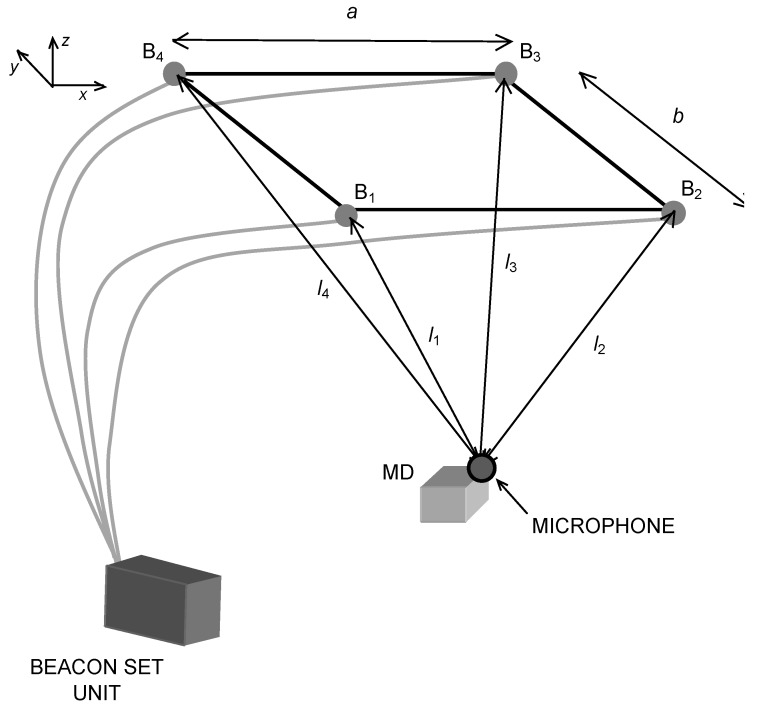
System architecture. The Beacon Set Unit emits the ultrasonic chirp signals through the four beacons B_1_, B_2,_, ..., B_4_; the microphone onboard the MD receives four ultrasonic signals and calculates its own position. The beacons belong to the same circuit and are intrinsically synchronized with each other.

The proposed system consists of four coplanar emitters, placed at the corners of a rectangle of sides *a* and *b*, designed to be positioned on the ceiling of a room (see Figure 1). At first, only an MD is considered, however in the following our considerations are extended to any number of MDs.

**Figure 2:**
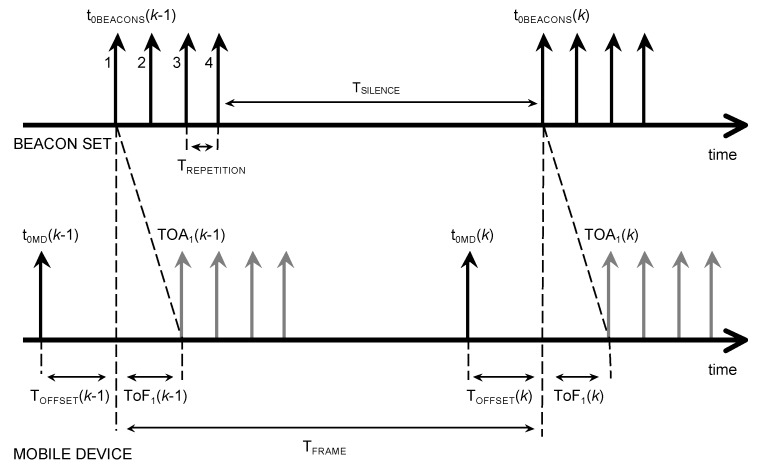
Time diagram for the Beacon Set and the mobile device (not in scale): The four beacons emit ultrasonic signals in a predefined sequence (i.e., 1, 2, 3, 4, T_SILENCE_, 1, 2, 3, 4…) starting from the time t_0BEACONS_, and the time interval between emissions is T_REPETITION_ (see Figure 2). The duration of each ultrasonic emission is T_EMISSION_ (not displayed) < T_REPETITION_.

The four beacons emit ultrasonic signals in a predefined sequence (i.e., 1, 2, 3, 4, T_SILENCE_, 1, 2, 3, 4…) starting from the time t_0BEACONS_, and the time interval between emissions is T_REPETITION_ (see Figure 2). Each emission duration is T_EMISSION_ < T_REPETITION_. The beacons belong to the same circuit and are intrinsically synchronized with each other. The sequence of the four signals is repeated in identical frames emitted at regular time intervals of duration T_FRAME_ (frame repetition time). Chirps are the simplest and more suitable ultrasonic signals that allow to take full advantage of correlation-based ranging techniques. Anyhow, the following reasoning applies to any type of signal that is able to provide a ranging with the required accuracy.

The MD records the ultrasonic signal coming from the Beacon Set for a duration time of T_FRAME_, according to its internal timer.

Therefore, two distinct periodic processes can be considered: (1) The ultrasonic emission frame repetition which starts at t_0BEACONS_; (2) the listening window which starts at t_0MD_ (see Figure 2). They are repeated with equal periods but with different starting times. There is, therefore, a lag or time offset between the two processes T_OFFSET =_ t_0BEACONS_- t_0MD_ that is unknown.

Typically, one of the synchronization techniques known in the literature is used to estimate T_OFFSET_, for example Reference Broadcasting [37,38], which however require additional hardware (wires, RF, etc.) and protocols. On the contrary, here an innovative solution completely devoid of synchronization hardware that allows the recovery of T_OFFSET_ is proposed.

The MD knows in advance the emitted signal, which is a linear chirp, in order to be able to process it and to obtain information on the arrival time. A very effective processing is the cross-correlation between the received chirp signal and a copy of the chirp stored in advance in the MD, which is used below. Digital cross-correlation shows high accuracy and, in general, good acoustical noise immunity in estimating TOA [39]. In short, the received acoustical signal is properly sampled and converted from analog to digital. The resulting numerical array of samples S is cross-correlated with the digital reference signal R, previously stored in the memory of the MD. The maximum of the cross-correlation indicates the point in time where S and R are best aligned. The lag τ, or inter-signals displacement, corresponding to the cross-correlation peak is proportional to the TOF, or in the absence of synchronization, proportional to the TOA referred to the local clock.

Since the MD does not know the time of emission of each ultrasonic signal, it is not able to estimate the TOF, but only the TOAs of each signal referred to the local clock, which are TOA_1_, TOA_2_, TOA_3_, TOA_4_, respectively. From these, three TDOA *dt_j_* (*j* = 1, 2, 3) are obtained:

*dt*_1_ = TOA_2_ − TOA_1_

*dt*_2_ = TOA_3_ − TOA_1_ (1)

*dt*_3_ = TOA_4_ − TOA_1_

Knowing the propagation velocity of the sound wave in the air *c_air_*:

(2) $$c_{air}=331.5\sqrt{1+\frac{T}{273.15}},$$

the range differences *d_j_* (*j* = 1, 2, 3) from the time differences are calculated as:

*d*_1_ = *dt_1_⋅c_ai_*_r_ = *l*_2_ − *l*_1_

*d*_2_ = *dt_2_⋅c_air_* = *l*_3_ − *l*_1_ (3)

*d*_3_ = *dt_3_⋅c_air_* = *l*_4_ − *l*_1_,

where *l*_1_, *l*_2_,…*l*_4_ are the distances between the MD and the four beacons. In what follows, it is assumed that, as usual, in a room the air flows and temperature gradient are kept under control for reasons of well-being of the occupants of the room. Residual fluctuations have very small effects on positioning accuracy. Alternatively, methods based on direct or indirect measurement of the speed of sound along the propagation path (see e.g., [40]) can be adopted.

If necessary, in environments with fast temperature variations, a temperature sensor for direct compensation can be included in the MD onboard equipment. From (3), using the intersection of hyperboloids, the coordinates (*x*, *y*, *z*) of the MD are calculated:

(4) $$\{\begin{array}{c} d_{1}=l_{2}-l_{1}=\sqrt{{\left(x-x_{RP2}\right)}^{2}+{\left(y-y_{RP2}\right)}^{2}+{\left(z-z_{RP2}\right)}^{2}}-\sqrt{{\left(x-x_{RP1}\right)}^{2}+{\left(y-y_{RP1}\right)}^{2}+{\left(z-z_{RP1}\right)}^{2}}\\ d_{2}=l_{3}-l_{1}=\sqrt{{\left(x-x_{RP3}\right)}^{2}+{\left(y-y_{RP3}\right)}^{2}+{\left(z-z_{RP3}\right)}^{2}}-\sqrt{{\left(x-x_{RP1}\right)}^{2}+{\left(y-y_{RP1}\right)}^{2}+{\left(z-z_{RP1}\right)}^{2}}\\ d_{3}=l_{4}-l_{1}=\sqrt{{\left(x-x_{RP4}\right)}^{2}+{\left(y-y_{RP4}\right)}^{2}+{\left(z-z_{RP4}\right)}^{2}}-\sqrt{{\left(x-x_{RP1}\right)}^{2}+{\left(y-y_{RP1}\right)}^{2}+{\left(z-z_{RP1}\right)}^{2}}, \end{array}$$

where *X_RPi_* = (*x_RPi_*, *y_RPi_*, *z_RPi_*) is the reference position of the *i*^th^ (*i* = 1, 2,…4) beacon.

The relative position of the MD with respect to the emitters, the distance between the emitters and the noise level greatly influence the accuracy of the solution. In particular, whenever the MD is located in points of space equidistant at least by two emitters, or in their proximity, the solutions of (4) are affected by errors whose entity can be significant, as shown later in Section 3.

Considering the arrangement of the four beacons at the corners of a rectangle of sides *a* and *b*, with coordinates *X_RP1_* = (*0*, *0*, *0*), *X_RP2_* = (*a*, *0*, *0*), *X_RP3_* = (*a*, *b*, *0*), *X_RP4_* = (*0*, *b*, *0*), respectively, the (4) can be rewritten as follows:

(5) $$\begin{array}{l} d_{1}=\sqrt{{\left(x-a\right)}^{2}+y^{2}+z^{2}}-\sqrt{x^{2}+y^{2}+z^{2}}=\sqrt{{\left(x-a\right)}^{2}+y^{2}+z^{2}}-l_{1}\\ d_{2}=\sqrt{{\left(x-a\right)}^{2}+{\left(y-b\right)}^{2}+z^{2}}-\sqrt{x^{2}+y^{2}+z^{2}}=\sqrt{{\left(x-a\right)}^{2}+{\left(y-b\right)}^{2}+z^{2}}-l_{1}\\ d_{3}=\sqrt{x^{2}+{\left(y-b\right)}^{2}+z^{2}}-\sqrt{x^{2}+y^{2}+z^{2}}=\sqrt{x^{2}+{\left(y-b\right)}^{2}+z^{2}}-l_{1}, \end{array}$$

and rearranging the terms:

(6) $$\begin{array}{l} {\left(d_{1}+l_{1}\right)}^{2}={\left(x-a\right)}^{2}+y^{2}+z^{2}\\ {\left(d_{2}+l_{1}\right)}^{2}={\left(x-a\right)}^{2}+{\left(y-b\right)}^{2}+z^{2}\\ {\left(d_{3}+l_{1}\right)}^{2}=x^{2}+{\left(y-b\right)}^{2}+z^{2}. \end{array}$$

Rewriting the first and third of the (6):

(7) $$\begin{array}{l} {\left(x-a\right)}^{2}+z^{2}={\left(d_{1}+l_{1}\right)}^{2}-y^{2}\\ {\left(y-b\right)}^{2}={\left(d_{3}+l_{1}\right)}^{2}-x^{2}-z^{2}, \end{array}$$

and replacing the (7) in the second of the (6), is obtained:

(8) $${\left(d_{2}+l_{1}\right)}^{2}={\left(d_{1}+l_{1}\right)}^{2}-y^{2}+{\left(d_{3}+l_{1}\right)}^{2}-x^{2}-z^{2}={\left(d_{1}+l_{1}\right)}^{2}+{\left(d_{3}+l_{1}\right)}^{2}-l_{1}{}^{2}.$$

Resolving the (8) for *l*_1_, finally is obtained:

(9) $$l_{1}=\frac{d_{2}^{2}-d_{1}^{2}-d_{3}^{2}}{2\left(d_{1}+d_{3}-d_{2}\right)}.$$

Equation (9) gives an estimate of *l*_1_ and from this, through the (2), of *l*_2_, *l*_3_, and *l*_4_. From *l*_1_, TOF_1_ = *l*_1_/c_air_ is obtained, and from this, knowing TOA_1_ = TOF_1_ + T_OFFSET_, T_OFFSET_ is finally estimated.

The accuracy of the current estimate of T_OFFSET_ from (9) strongly depends on the position of the MD with respect to the four beacons. The (9) produces bad estimates in some positions, where the denominator becomes very small or almost zero. In the space points set where $d_{1}+d_{3}-d_{2}=0$ there is an unlimited error and (9) is “blind”. Each T_OFFSET_ estimated directly from (9) is therefore affected by a considerable uncertainty or noise (see Figure 6).

It is important to note that T_OFFSET_ is the same for all the beacons, which belong to the same synchronous circuit, and, above all, T_OFFSET_ is the same for any position of the MD.

At each positioning operation, the obtained noisy value of T_OFFSET_ can be used to refine the estimate of the true value of T_OFFSET_.

As already mentioned in Section 1, there are two processes running concurrently, one in the Beacon Set and the other in the MD, which in principle are perfectly synchronous, where the difference between the initial times of the two clocks T_OFFSET_ is the only unknown factor.

If T_OFFSET_ were really constant, in order to obtain an accurate estimate of the T_OFFSET_, it would be enough to make an average of the noisy values of the rough T_OFFSET_ estimates coming from (9), provided that the noise has zero mean value. This is in fact the approach followed by [33], which used a moving average. In practice, however, this approach does not work well.

In fact, even if nominally the clocks of the beacon set and of the MD have the same frequency, in practice the frequencies of the two clocks differ for some part per million (ppm) and there is a clock drift over time. Considering commercial microcontrollers with a quartz clock, a typical value for clock total uncertainty is 100 ppm, depending on the temperature, manufacture, and aging of the clock components.

Clock drift, which is one of the components of the total uncertainty of a clock, produces remarkable effects. For example, if the clock frequency of our beacon set and MD systems is 10 MHz, and they differ by 50 ppm, after one second, in the worst case, the two clocks differ by 500 clock periods or 50 μs. Assuming a sound speed of 343 m/s, this difference produces a measurement error of 1.7 cm.

However, it is worth noting that, even starting from the unrealistically favorable hypothesis of initial synchronization between the two clocks, over the course of time an error of 1.7 cm is accumulated for every additional second of the system operation, and therefore after 60 s the error exceeds 1 m. The presence of clock drift is such that an event produced by the beacons at constant intervals is seen by the MD as happening at slowly increasing intervals over time. With a clock drift, the MD actually records an event that happens at the beacons at constant intervals, as if it happened at intervals that slowly increase over time. At MD, the emission constant time interval becomes a time ramp and the same happens to the T_OFFSET_. Therefore, T_OFFSET_(*k*) has to be estimated as a function of time, where *k* is a time index (see below).

It is possible to estimate the clock drift value through a ramp follower [41], under the hypothesis that the value of the drift, or the slope of the time ramp, remains reasonably constant or very slow varying during the time interval of the operations of the system:

(10) $$\begin{array}{l} e(k)=y(k)-ref(k)\\ \left[\begin{array}{c} x_{1}\left(k+1\right)\\ x_{2}\left(k+1\right) \end{array}\right]=\left[\begin{array}{cc} 1 & 0\\ 0.01 & 1 \end{array}\right]\left[\begin{array}{c} x_{1}\left(k\right)\\ x_{2}\left(k\right) \end{array}\right]+\left[\begin{array}{c} 0.01\\ 5\cdot 10^{-5} \end{array}\right]e(k)\\ y(k+1)=K\left[\begin{array}{cc} 1 & 1 \end{array}\right]\left[\begin{array}{c} x_{1}\left(k+1\right)\\ x_{2}\left(k+1\right) \end{array}\right], \end{array}$$

where *k* is the *k*th time step of *T_FRAME_* duration, i.e., the repetition time of the positioning operation, *ref*(*k*) is the noisy ramp to be followed, i.e., the current value of T_OFFSET_(*k*), *y*(*k*) = T^*^_OFFSET_(*k*) is the estimate of the true T_OFFSET_(*k*) after the noise rejection, *x*_1_(*k*) and *x*_2_(*k*) are the internal states of the ramp follower, and *K* is a constant parameter tuned by trial and error procedure, the smaller its value, the greater the noise rejection and convergence time of the ramp follower. Note that (10) is only one of the possible ways to estimate a ramp shaped T^*^_OFFSET_(*k*), however a thorough discussion on this issue is beyond the scope of this work.

It is worth noting that, if an output follower were applied to the time sequence of coordinates from (4), in order to filter out the jerks due to the numerically weak solution of the intersection of hyperboloids, its time filtering effect would make it difficult to reproduce abrupt variations well.

On the contrary, in this work, the ramp follower is applied to the parameter T_OFFSET_ which varies very slowly, obtaining the T^*^_OFFSET_. It is worth noting that (10) converges recovering the synchronization without having any prior information on the T_OFFSET_ and starting from the first value provided by (9). It is also possible to observe that Equations (9)–(10) do not introduce any constraint on the trajectory of the measured MD positions to estimate *l_1_* and T*_OFFSET_. In fact, (10) converges regardless of the trajectory followed by the MD.

The T^*^_OFFSET_(*k*) sequence thus obtained, although heavily filtered over time, allows to obtain the TOF(*k*)_i_ = TOA(*k*)_i_ + T^*^_OFFSET_(*k*) sequence and therefore to use the intersection of spheres (12)–(13), which produces much more accurate results than the intersection of hyperboloids (4), therefore overcoming the issue of noisy results from (4). Ultimately, this approach allows trajectories to be tracked with reduced noise even in the presence of abrupt variations.

The T*_OFFSET_(*k*) estimate allows finding the estimates l^*^_i_(*k*) through the following:

l^*^_1_(*k*) = [TOA_1_(*k*) + T^*^_OFFSET_(*k*)]·*c_air_*

l^*^_2_(*k*) = [TOA_2_(*k*) + T^*^_OFFSET_(*k*)]·*c_air_* (11)

l^*^_3_(*k*) = [TOA_3_(*k*) + T^*^_OFFSET_(*k*)]·*c_air_*

l^*^_4_(*k*) = [TOA_4_(*k*) + T^*^_OFFSET_(*k*)]·*c_air_*.

Equation (11) allows calculating the estimate of the position of the MD at the *k*th positioning frame through the simplified spherical equations intersection solution [22,29]:

(12) $$\{\begin{array}{l} l_{1}^{*2}=x^{2}+y^{2}+z^{2}\\ l_{2}^{*2}={\left(x-a\right)}^{2}+y^{2}+z^{2}\\ l_{3}^{*2}={\left(x-a\right)}^{2}+{\left(y-b\right)}^{2}+z^{2}\\ l_{4}^{*2}=x^{2}+{\left(y-b\right)}^{2}+z^{2}. \end{array}$$

By picking in all combinations three sphere equations at a time from the available four (12), four sets of coordinates for the position of the MD are obtained:

(13) $$\begin{array}{l} x_{1}=\frac{l_{1}^{*2}-l_{2}^{*2}+a^{2}}{2a}\\ y_{1}=\frac{l_{1}^{*2}-l_{4}^{*2}+b^{2}}{2b}\\ z_{1}=\sqrt{|l_{1}^{*2}-x_{1}^{2}-y_{1}^{2}|,} \end{array}\begin{array}{l} x_{2}=\frac{\;l_{1}^{*2}-l_{2}^{*2}-a^{2}}{2a}+a\\ y_{2}=\frac{l_{2}^{*2}-l_{3}^{*2}+b^{2}}{2b}\\ z_{2}=\sqrt{\left|l_{2}^{*2}-{\left(x_{2}-a\right)}^{2}-y_{2}^{2}\right|} \end{array}\;\begin{array}{l} x_{3}=\frac{l_{4}^{*2}-l_{3}^{*2}-a^{2}}{2a}+a\\ y_{3}=\frac{l_{2}^{*2}-l_{3}^{*2}-b^{2}}{2b}+b\\ z_{3}=\sqrt{|l_{3}^{*2}-{\left(x_{3}-a\right)}^{2}-{\left(y_{3}-b\right)}^{2}|,} \end{array}\begin{array}{l} x_{4}=\frac{\;l_{4}^{*2}-l_{3}^{*2}+a^{2}}{2a}\\ y_{4}=\frac{l_{1}^{*2}-l_{4}^{*2}-b^{2}}{2b}+b\\ z_{4}=\sqrt{|l_{4}^{*2}-x_{4}^{*2}-{\left(y_{4}-b\right)}^{2}|.} \end{array}$$

with *z_i_* ≥ 0 (*i* = 1, 2...4).

Finally, the position of the MD can be calculated as an average of the four positions computed by (13), making it more robust against small and unbiased errors on the estimates of the four distances:

(14) $$\{\begin{array}{c} x=\frac{1}{4}\sum_{h=1}^{4}x_{h}\\ y=\frac{1}{4}\sum_{h=1}^{4}y_{h}\\ z=\frac{1}{4}\sum_{h=1}^{4}z_{h}. \end{array}$$

**Figure 3:**
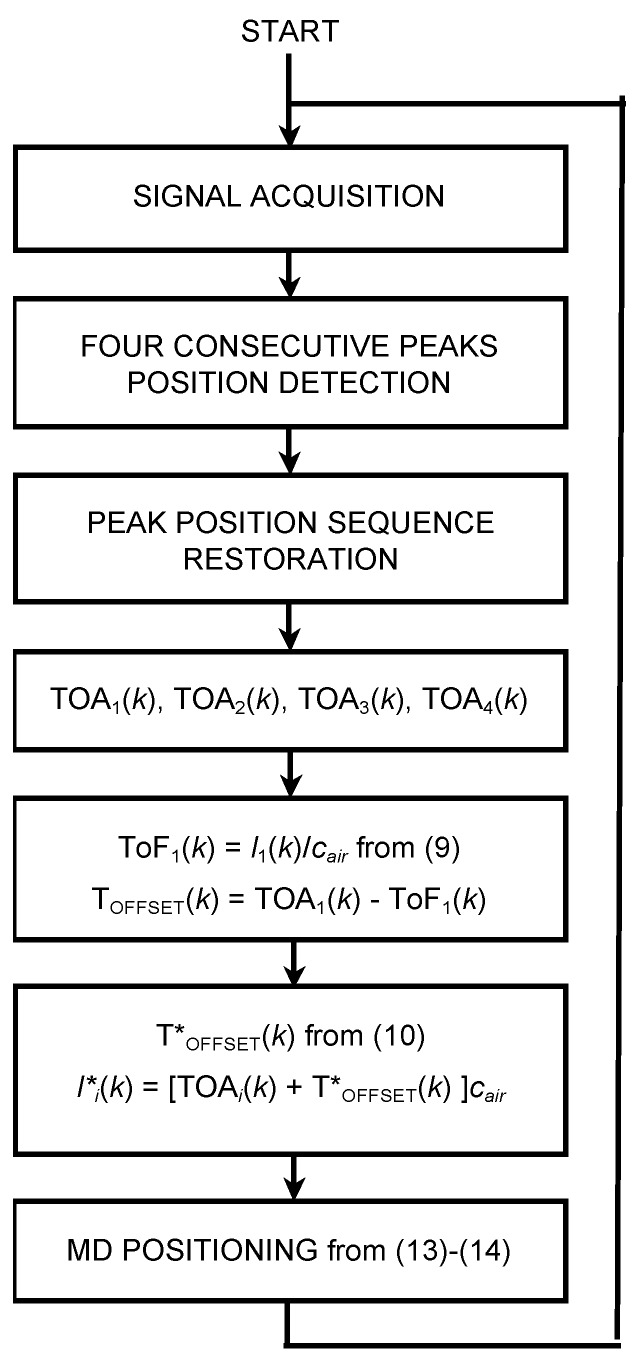
Block diagram of the proposed positioning algorithm that operates in an infinite loop after initializing T_OFFSET_(0) = 0 and T*_OFFSET_(0) = 0. Peak position sequence restoration at the third step of the algorithm is required when the incoming arrival instants (i.e., the detected peaks) do not appear in the natural sequence that occurs for certain values of T_OFFSET_.

A block diagram of the proposed positioning algorithm, as executed onboard the MD, is shown in Figure 3. 

At the start, T_OFFSET_(0) and T*_OFFSET_(0) are set to zero. Subsequently, the algorithm works as an infinite loop. During the signal acquisition phase, the ultrasonic signal is received, sampled, and recorded for a period of time suitable for surely acquiring all four ultrasonic emissions considering the duration of the twitter used. The recording duration is therefore longer than the T_FRAME_ used. The details are described in Section 3. The successive step includes the cross-correlation between the recorded signal and a copy of the expected signal, previously stored in the MD memory. In this way, four sharp consecutive peaks are obtained. The positions, or lags, of the peaks in the computed cross-correlation vector are proportional to the four TOAs of the four ultrasonic signals travelling from the emitter to the MD. 

In our system, the emission sequence is “1, 2, 3, 4” followed by a suitable “silence window”, much longer than the duration of the chirp (see Figure 2). For always increasing (or decreasing) values of T_OFFSET_ frame by frame, this order may appear altered in reception. For example, due to the clock drift, after a certain operation time, the sequence “4, silence window, 1, 2, 3” can be received instead of the expected sequence “1, 2, 3, 4, silence window”. Furthermore, it is clear that T_OFFSET_ has a cyclic behavior, since every time it exceeds the T_FRAME_ value, it returns to zero, wrapping around the value T_FRAME_ as in the modulus operation. The correct sequence of events is restored at the receiver periodically in the third step of the algorithm by considering each intervened T_OFFSET_ reset along time.

The system is conceived for use with non-stationary MDs. In fact, in this case the major part of the noise on the TO estimate is non-stationary too and therefore it is properly averaged and rejected.

The single positioning operation has minimal computational impact: Evaluation of 12 equations (see Equation (13)), averaging of (14), and single update of the tracker (see Equations (9) and (10)) for each computed position.

## 3. Algorithm Simulation and Experimental Results

The verification of the theoretical results is initially made with the MATLAB code simulation of a system suitable to provide positioning in a typical office or home room, and subsequently with the realization and characterization of an experimental prototype.

### 3.1. System Simulation Setup and Results

The following simulation shows that the proposed algorithm converges in the estimate of the true T_OFFSET_ at the accuracy level that can be achieved with the given sampling frequency F_S_. The beacon system is fixed at the center of the ceiling of a 4 × 4 × 3 m^3^ room. The beacons are fixed at the corners of a square having side *a* = 50 cm, so as to constitute an element that can easily be integrated into a typical room ceiling panel.

The simulation computations start from the knowledge of the four TOAs, which are detected in the correlation between received signal and signal stored in memory with uncertainty Δ_TOA_ [18,38]. In the following simulations, F_S_ = 1/T_S_ = 192 kS/s (F_S_ = 192 kHz) and Δ_TOA_ = ± T_S_/2 have been set. 

Assuming the sound speed 343 m/s, the time interval of T_S_ corresponds to a space interval of 1.8 mm. A random value for the starting T_OFFSET_(0) and a Beacon Set-MD relative clock drift of 200 ppm has been assumed. 

In the presence of clock drift, we have:

T_OFFSET_(*k*) = T_OFFSET_(0) + Δ_CLOCK_⋅*k*, (15)

where Δ_CLOCK_ = 25 μs is computed taking into account that a 200 ppm clock drift applies to an external 32768 Hz crystal, from which the microprocessor clock is derived, and T_FRAME_ is 0.125 s. 

**Figure 4:**
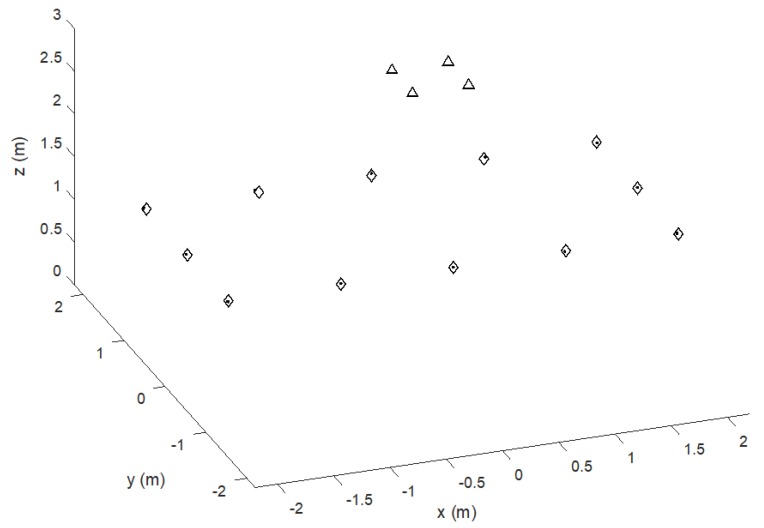
Simulated trajectory of the moving MD (diamonds) and estimated positioning (dots): Last 12 positioning frames. Beacons are indicated by triangles. The positioning error, expressed as Euclidean distance between reference and estimated points *e_ED_*, is shown by the last 12 values of Figure 5.

**Figure 5:**
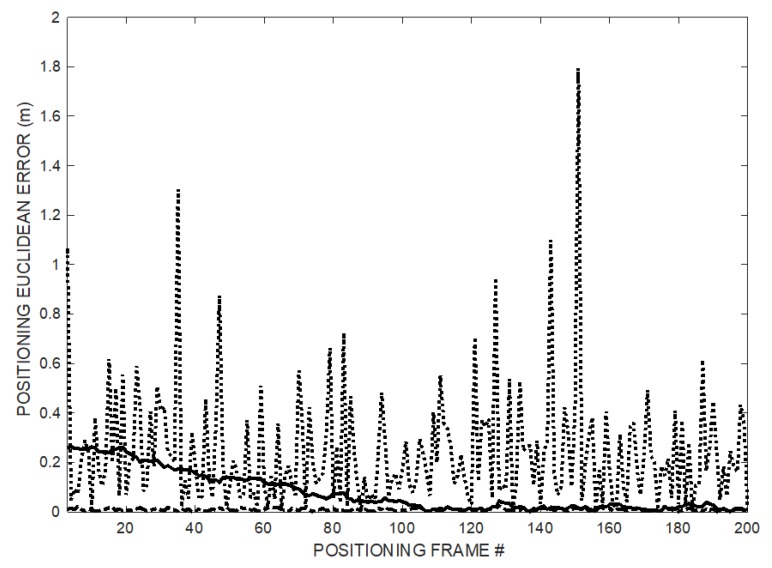
Decreasing positioning error *e_ED_* over 200 successive positioning frames: TDOA (dotted line), synchronized TOF (dash-dot line), and proposed method (thick solid line).

In the simulation, the MD moves along a preset trajectory following the sides of a 2 × 4 m^2^ rectangle, with 12 positioning measures or frames every 1 m along the way (se Figure 4) and the T_FRAME_ is 0.125 s (i.e., frame rate 8 Hz). The rectangular trajectory is repeated for a total of 200 positioning frames to show the convergent behavior of the T_OFFSET_ recovery process. Figure 4 shows the estimated trajectory compared to the true one indicated by the last 12 position estimates, i.e., after algorithm convergence. The overall 3D positioning error at coordinates (*x’*, *y’*, *z’*) is given by the Euclidean distance $e_{ED}=\;\sqrt[{2}]{{\left(x^{\prime }-x\right)}^{2}+{\left(y^{\prime }-y\right)}^{2}+{\left(z^{\prime }-z\right)}^{2}}$ between each ground truth point at coordinates (*x’*, *y’*, *z’*) and the estimated one at coordinates (*x*, *y*, *z*). Figure 5 reports the *e_ED_* behavior along the 200 positioning frames. The last 12 values refer to the positioning shown in Figure 4, after the convergence of the process from successive application of (9) and (10) starting from no prior information on the T_OFFSET_ value.

The positioning error *e_ED_* is shown in Figure 5: The error obtained using the proposed method decreases over time as ramp follower converges (thick solid line) approaching the small error achievable by the synchronized TOF positioning (dash-dot line), while the error obtained using TDOA remains large (dotted line).

**Figure 6:**
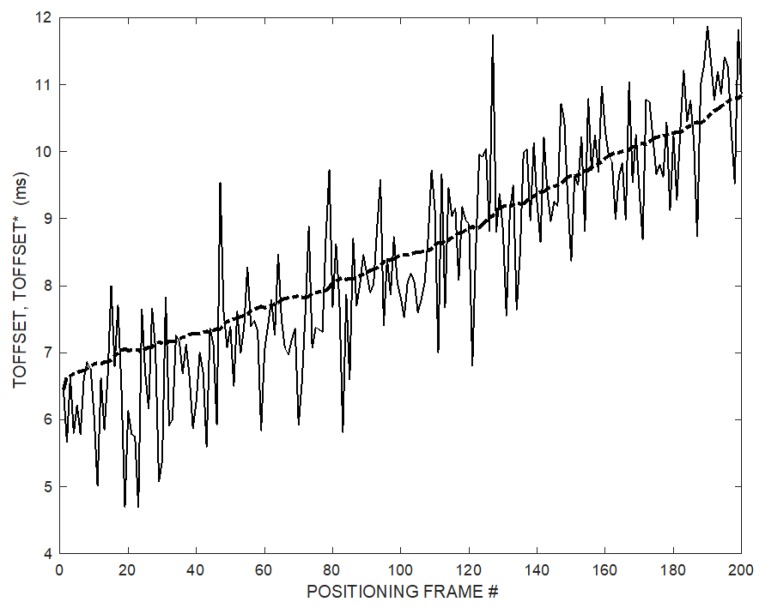
Instantaneous T_OFFSET_ (solid line) and estimated T^*^_OFFSET_ (dash-dot line) while the MD is continuously moving on its trajectory. T_OFFSET_ is affected by a relevant noise. T^*^_OFFSET_ is the output of the ramp follower (10) and converges without initial guess or prior information.

Figure 6 shows the instantaneous values of T_OFFSET_, and its over time estimate T^*^_OFFSET_ using the ramp follower (10) with *K* = 2.5.

**Figure 7:**
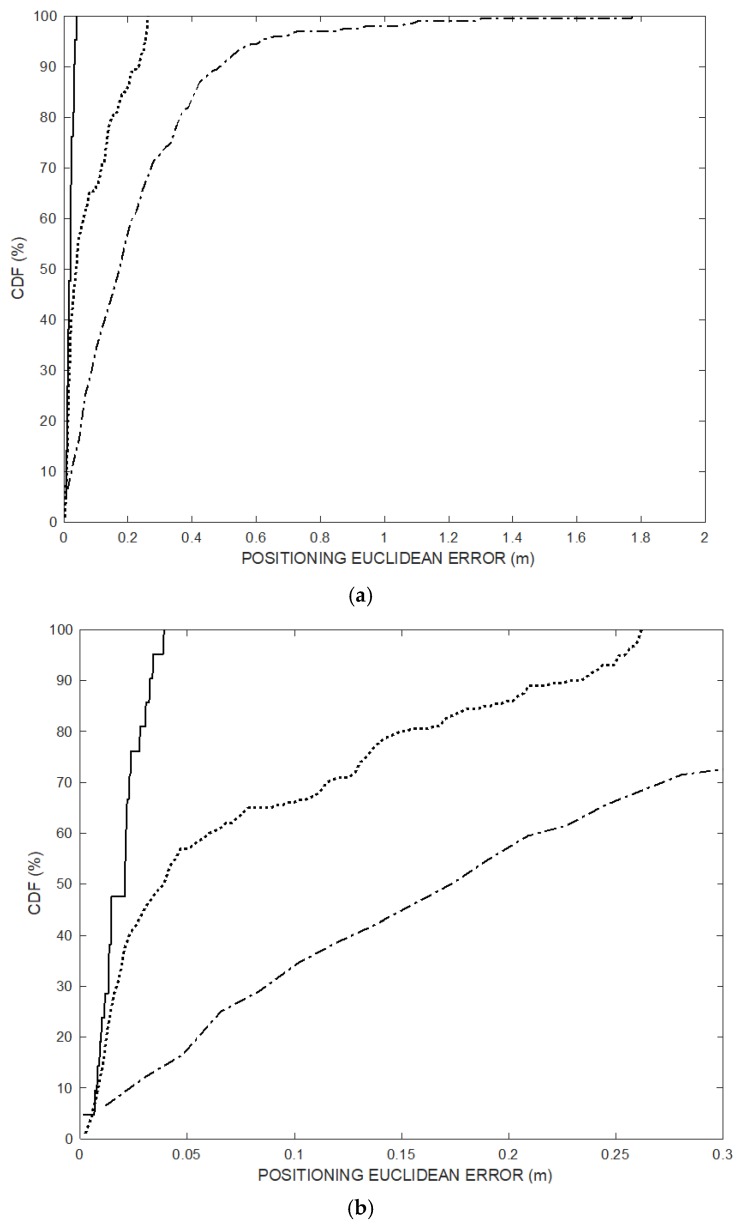
(**a**) Cumulative error distributions (percent of readings with error less than the value of a given abscissa) of the positioning over the 200 positioning frames (dotted line) and of the last 20 trajectory points (solid line) after convergence transient, compared to CDF of the TDOA technique (dash-dot line). (**b**) X-axis zoomed portion. The positioning Euclidian error of the last 20 points obtained by the proposed method is below 5 cm.

Figure 7 shows the cumulative error distribution (CDF) of the simulated positioning data set of Figure 4 over the 200 positioning frames (dotted line) and of the last 20 trajectory points (solid line) after convergence transient, compared to CDF of the TDOA technique (dash-dot line). The positioning Euclidian error of the last 20 points obtained by the proposed method is below 5 cm.

### 3.2. Experimental System Realization

In general, it is not trivial to know the true position of an MD at any time to compare it with the estimated one, especially when points belonging to any 3D trajectory are considered. However, a reference position set is necessary to characterize and validate a 3D positioning system. Therefore, a prototype PC based positioning system was developed, capable of providing both a reference position and the one estimated with the proposed method in order to make the comparison.

The system consists of a PC, a Data Emission/Acquisition Board, an ultrasonic Emitter Set, and microphone. It emulates the beacons/MD system and is capable of executing both the asynchronous algorithm described in the block diagram of Figure 3 and the synchronous positioning described in detail in [18]. Briefly, the system described in [18] provides the position of an MD using a technique of intersection of spheres based on four TOFs calculated by means of emissions and reception of ultrasonic signals synchronized through a suitable RF channel. 

The present prototype emulates the positioning system [18] step by step and shows the same positioning performances. In fact, it emits the same signals, carries out the same operations on the same input data, and produces positioning results identical to those of the system reported in [18].

At the same time, this system performs the steps of the algorithm proposed here (see Figure 3) to also calculate the MD trajectory without taking into account the synchronization, i.e., asynchronously. Then, comparing the positions obtained separately with the two methods, synchronous and asynchronous, the additional error committed specifically by the proposed synchronization recovery method is estimated.

However, in the PC/Board system there is no clock drift since the process of emission and reception of the ultrasonic signal are inherently synchronous as they are managed by the Data Emission/Acquisition board processor itself. Therefore, in calculating the position with the asynchronous method, a drift of 200 ppm has been added in order to simulate the clock drift that affects the asynchronous applications of the real world, plus a starting time offset of 7 ms.

The different components forming our laboratory positioning system setup are listed below:

Processing unit: A PC (Intel Core 2 Duo, 3.06 GHz, 8 GB RAM) is employed as the central processing unit of the positioning system. It runs a code written in MATLAB (The MathWorks, Inc.) for ultrasonic signal generation and for acquiring, storing, and analyzing the signals received by the single microphone.

**Figure 8:**
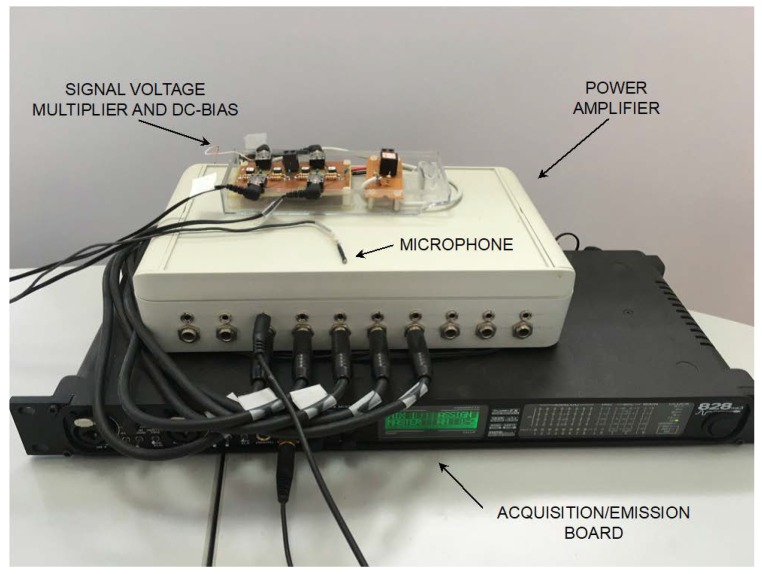
Data emission/acquisition board, power amplifier, signal voltage multiplier, and 200 V DC-bias circuitry, and wired miniature microphone.

Data Emission/Acquisition Board: MOTU 828 mk3 (MOTU, Cambridge, Massachusetts, USA). It is provided with ten analog inputs and outputs that can operate at sample rates up to 192 ksamples/s (see Figure 8). The PC connection is realized through FireWire. Four outputs and one input are employed in our experimental setup. A linear up-chirp in the bandwidth 30–50 kHz is employed. The chirp signal, composed of 512 samples at 192 ksamples/s, is Hanning windowed to avoid audible “clicking”, while the emission sequence shows T_FRAME_ = 125 ms, T_EMISSION_ = 2.66 ms, T_REPETITION_ = 5 ms, and T_SILENCE_ = 109.34 ms (see Figure 2).

Ultrasonic power amplifier and emitters: The generated chirps are emitted in sequence through four MOTU output channels. They are voltage amplified and fed to a four-channel Class AB MOSFET power amplifier. Each chirp signal level is further raised to 300 Vp-p in each of the four output channels with a signal voltage multiplier realized using the miniaturized 1:100 coil transformer LPR6235-752S (Coilcraft, Glasgow, UK) and fed to four ultrasonic transducers Series 7000 Electrostatic Transducer (SensComp Inc., Livonia, MI, USA) [42]. The capacitive transducers are DC-biased by 200 V supplied by the ultra-miniature DC to HV DC Converter Q02-5-R (EMCO High Voltage Corp., Sutter Creek, CA, USA).

Since Series 7000 transducers have not been designed for such application and their emission cone is not wide enough to cover the intended volume, they were adapted according to the extensive discussion in [18]. Their emission cone half angle (far field) was widened from 24.7° up to 80.95° at 50 kHz by reducing the aperture diameter down to 8.5 mm with a suitable mask.

**Figure 9:**
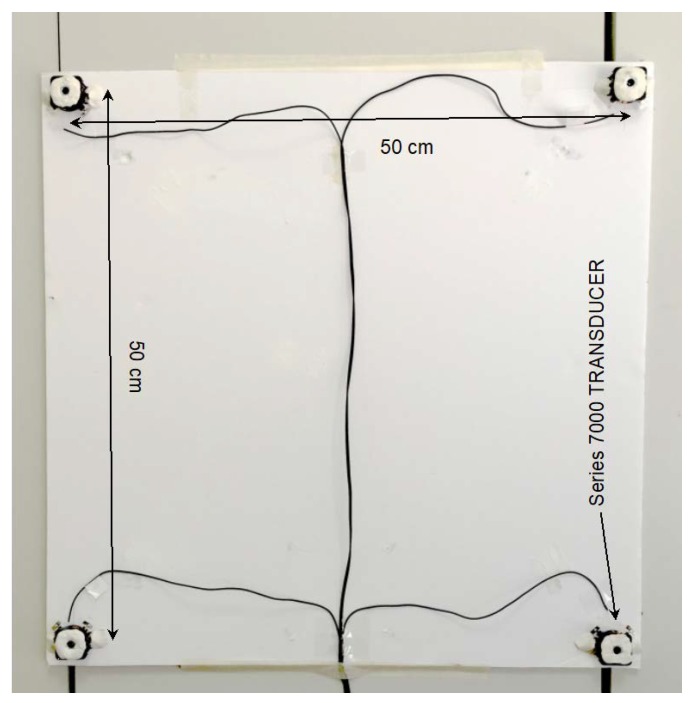
Beacon Set Unit: 52 × 52 cm^2^ panel hosting four capacitive SensComp Series 7000 transducers. Their emission cone half angle (far field) is widened from 24.7 up to 80.95° at 50 kHz by reducing the aperture diameter down to 8.5 mm with an aperture mask made of white moldable material.

The Beacon Set Unit components are mounted on a square 52 × 52 cm^2^ panel. The four transducers are placed at the corners of a 50 × 50 cm^2^ square, face up toward the room volume (see Figure 9). 

Mobile Device: Here, the MD is mimicked by a wired miniature microphone, amplified, and sampled by the MOTU at 192 ksamples/s (F_S_ = 192 kHz). The microphone is an FG-6163 (Knowles Acoustics, Itasca, Illinois, USA), a micromachined condenser microphone encapsulated in a cylindrical package, 2.6 mm length and diameter, 0.79 mm acoustical receiver window diameter, and 80 mg weight (see Figure 8). This microphone is the same one that equips the MD described in [18]. 

### 3.3. Experimental Results

The experimental trajectory was obtained by manually moving a microphone along a random 3D path. The microphone is continuously moved at a moderate speed, such as a normal human gesture (< 1 m/s). During experiments, the sound velocity was assumed to be constant at 344.6 m/s (T = 22.0 °C).

The four emitters driven by the power amplifier fed by the acquisition/emission board emit in sequence the ultrasonic signals at the rate of 8 Hz (1/T_FRAME_). This positioning rate is actually limited by the computational power of the employed PC. 

**Figure 10:**
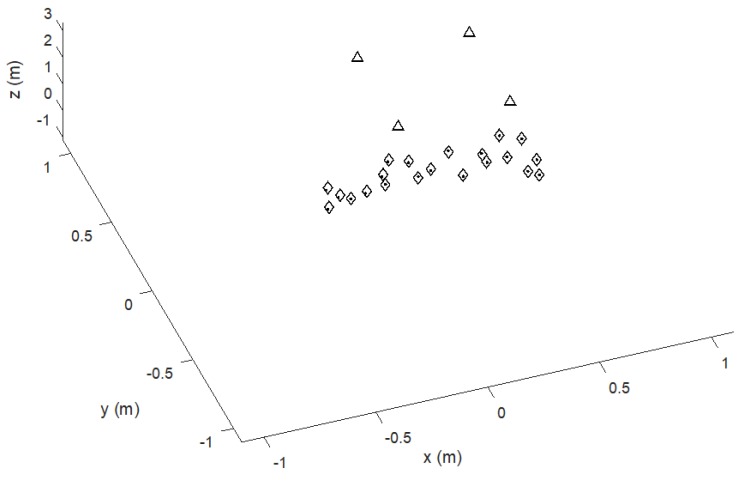
Experimental trajectory of the moving microphone obtained with synchronized range measurements (diamonds) and trajectory obtained using the proposed synchronization recovery method (dots): Last 20 positioning frames. Beacons are indicated by triangles.

The ultrasonic signal is received through the microphone, conditioned, amplified, and recorded by the acquisition/emission board. The recorded signal is sent to the PC where the MATLAB code performs both synchronous and asynchronous positioning, estimating the T_OFFSET_ along the successive positioning frames. After the convergence of the synchronization recovery process, the microphone coordinates measured by the system are in good agreement with the ones measured using the synchronism information (see Figure 10).

Since the reference trajectory and the trajectory estimated with the proposed asynchronous technique are computed starting from the same ultrasonic data, the difference between them is exclusively due to the errors in the T_OFFSET_ estimation. Microphone absolute positioning uncertainty is instead mainly due to the TOA time quantization error propagation, deeply discussed in our previous work [18].

**Figure 11:**
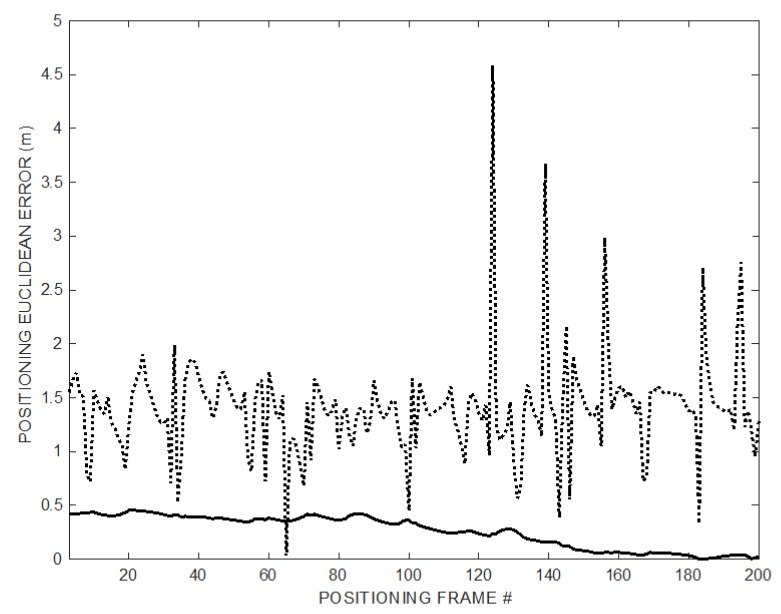
Experimental decreasing positioning error *e_ED_* over 200 successive positioning frames: TDOA (dotted line) and proposed method (solid line).

In Figure 11, the experimental results of the positioning operations for all the points on the trajectory are plotted. In particular, the positioning error is computed as the point-to-point Euclidian distance of the trajectory obtained using the synchronization recovery method from the trajectory obtained using the synchronization information.

**Figure 12:**
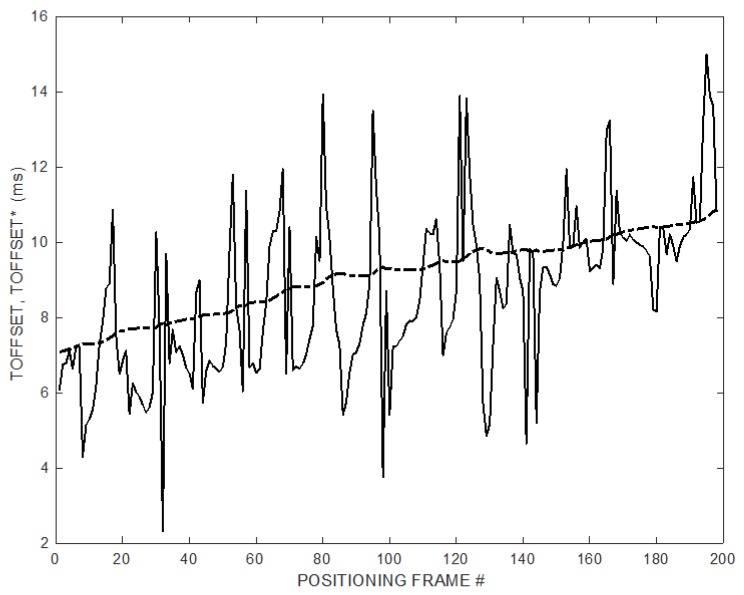
Experimental instantaneous T_OFFSET_ (solid line) and estimated T^*^_OFFSET_ (dash-dot line) while the microphone is moved along its trajectory. T_OFFSET_ is affected by a relevant noise. T^*^_OFFSET_ is the output of the ramp follower (10).

Figure 12 shows the experimental instantaneous values of T_OFFSET_, and its estimate over time T^*^_OFFSET_ using the ramp follower (10) with *K* = 2.5.

**Figure 13:**
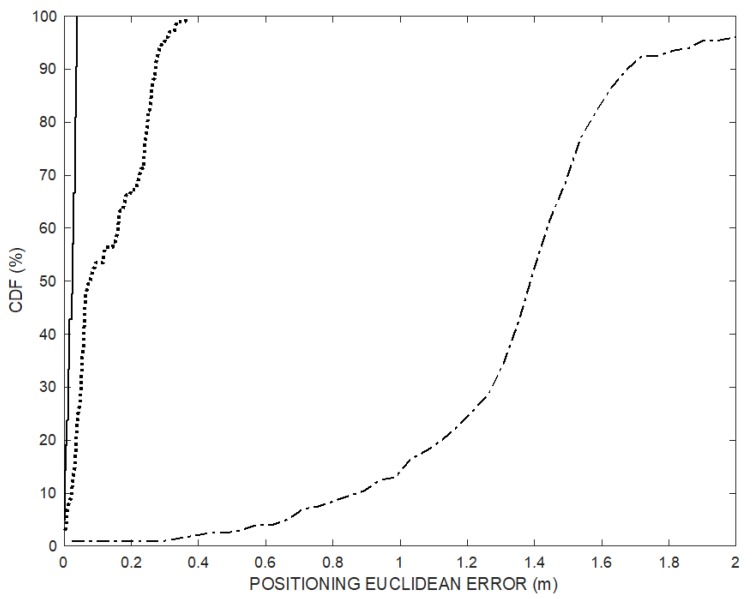
Cumulative error distributions (percent of readings with error less than the value of a given abscissa) of the positioning over the 200 positioning frames (dotted line) and of the last 20 trajectory points (solid line) after convergence transient, compared to CDF of the TDOA technique (dash-dot line). The positioning Euclidian error of the last 20 points obtained by the proposed method is below 5 cm.

Figure 13 shows the cumulative error distribution (CDF) of the experimental positioning data set of Figure 11 over the 200 positioning frames (dotted line) and of the last 20 trajectory points (solid line) after convergence transient, compared to CDF of the TDOA technique (dash-dot line). The positioning Euclidian error of the last 20 points obtained by the proposed method after convergence is below 5 cm. 

### 3.4. Error Propagation Remarks

Although the causes of error are several, here for simplicity only the effect of quantization error is presented (see for further details [18]). The quantization error has a uniform distribution and, from an engineering point of view, it is useful to know the worst-case error, which is the maximum error that can be made on each coordinate.

Briefly, considering the functional relationship *y* = *f*(*x*_1_, *x*_2_,…*x*_k_), small changes Δ*x*_1_ in *x*_1_, Δ*x*_2_ in *x*_2_,…, Δ*x*_k_ in *x*_k_, all propagate to produce a worst-case small change Δ*y* in *y* in the following manner:

(16) $$\Delta f≅{\sum_{j=1}^{k}\left|\frac{\partial f}{\partial x_{j}}\right|}_{x_{j}=x_{j0}}\Delta x_{j}.,$$

The use of the partial derivative module takes into account the fact that in the worst case the contributions relating to each of the independent variables are added together.

Equation (16) is used when it is not given to repeat a sufficiently high number of measures (>10) to estimate the average quadratic error. We are considering an MD that does not stay fixed in space, so that generally the measurement at a given spatial point is single. Equation (16) is actually an approximation, since any higher order terms such as (Δ*x*_1_)^2^ have been set to zero, however, it is sufficiently accurate for most purposes.

For the error propagation analysis, it is sufficient to consider the positioning with three emitters, the fourth one employed to determine the validity of the measure and reduce the location error through the mean. By applying (16) to the second group of (13), it is therefore obtained:

(17) $$\begin{array}{l} \Delta x≅\left|\frac{\partial x}{\partial l_{1}}\right|\Delta l_{1}+\left|\frac{\partial x}{\partial l_{2}}\right|\Delta l_{2}=\left|\frac{l_{1}}{a}\right|\Delta l_{1}+\left|\frac{l_{2}}{a}\right|\Delta l_{2}\\ \Delta y≅\left|\frac{\partial y}{\partial l_{1}}\right|\Delta l_{1}+\left|\frac{\partial y}{\partial l_{2}}\right|\Delta l_{2}=\left|\frac{l_{2}}{b}\right|\Delta l_{2}+\left|-\frac{l_{3}}{b}\right|\Delta l_{3}\\ \Delta z≅\left|\frac{\partial z}{\partial l_{1}}\right|\Delta l_{1}+\left|\frac{\partial z}{\partial l_{2}}\right|\Delta l_{2}+\left|\frac{\partial z}{\partial l_{3}}\right|\Delta l_{3}=\left|\frac{-l_{1}x}{az}\right|\Delta l_{1}+\left|\frac{l_{2}}{z}\left(\frac{x}{a}-\frac{y}{b}+2\right)\right|\Delta l_{2}+\left|\frac{l_{3}y}{bz}\right|\Delta l_{3}, \end{array}$$

where Δ*l*_j_ is the quantization error of the measurement of the Beacon-MD distance. In the worst case, Δ*l*_j_ is equal to the half quantization interval, i.e., Δ*l*_j_ = *v*T_s_/2, where *v* is the speed of the sound in air and *T_s_* the sampling period. In (17), the resulting values must be considered values that cannot under any circumstances be exceeded, and not as standard deviations. However, in practice, the typical values may be smaller but reasonably of the same order of magnitude.

Δ*x* and Δ*y* are proportional to the Beacon-MD distance, while Δ*z* explicitly depends on the MD *x* and *y* coordinates. In our experimental setup, with *a* = *b* = 0.5 m and Δ*l _i_* ≤ 0.9 mm (i.e., half range quantum Δ*l*_j_ = *v*T_s_/2, with F_S_ = 192 kHz) and considering that the geometrical center of the system is (0.25, 0.25, 3), at the furthest and less favorable point in the 4 × 4 × 3 m^3^ room *P* = (−1.75, 2.25, 0) using (17) $\Delta P_{MAX}=\sqrt{\Delta x^{2}+\Delta y^{2}+\Delta z^{2}}=2.94\;cm.$

Δ*P_MAX_* must be regarded as upper bound of the real error, which occurs only in the worst case when all the Δ*l_i_* (*i* = 1, 2, 3) assume their maximum value at the same time. Equation (17) shows that to obtain a positioning accuracy of the order of centimeters, measurement accuracy of the order of millimeters is required. Moreover, it shows that the error magnitude is approximately inversely proportional to the length of the sides *a* and *b* of the rectangle formed by the Beacons.

**Figure 14:**
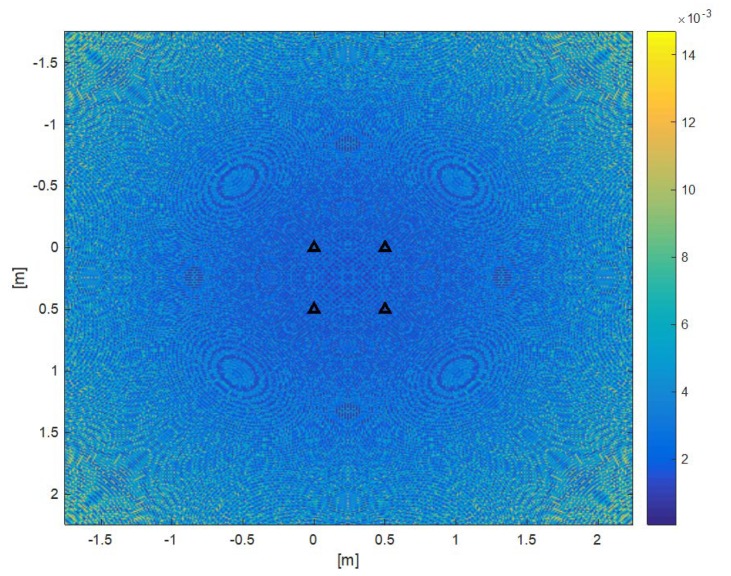
Simulation quantization error computed in a grid of points of plane z = 1.5 m, and by considering in sequence *a* = *b* = 0.5 m. The maximum error magnitude is 14.7 mm. Same error shape with *a* = *b* = 1.5 and 0.25 m, but with maximum error about 5.1 and 32.0 mm, respectively. Triangles show beacon positions. The error magnitude is of the same order but less than the value provided by (17), which is in fact the upper bound of the real error. The quadrangular symmetry of the Beacon Set produces an error shape with the same symmetry without blind or poor accuracy points.

The positioning error $\Delta P=\sqrt{{\left(x_{TRUE}-x_{ESTIMATED}\right)}^{2}+{\left(y_{TRUE}-y_{ESTIMATED}\right)}^{2}+{\left(z_{TRUE}-z_{ESTIMATED}\right)}^{2}}$ due to the quantization error is shown in Figure 14. It is computed in a grid of points of plane z = 1.5 m (Beacon Set at z = 3) with *a* = *b* = 0.5 m, with maximum error about 14.7 mm. Same error shape with *a* = *b* = 1.5 and 0.25 m, but with maximum error about 5.1 and 32.0 mm, respectively. 

The error magnitude is of the same order, but lower, than the value provided by (17), which is in fact the upper bound of the real error. In particular, the quadrangular symmetry of the Beacon Set produces an error shape with the same symmetry, as shown in Figure 14. It is worth noting that there are no blind or poor accuracy points, since the positioning is performed with the spherical equations (13), once T*_OFFSET_ has been estimated.

**Figure 15:**
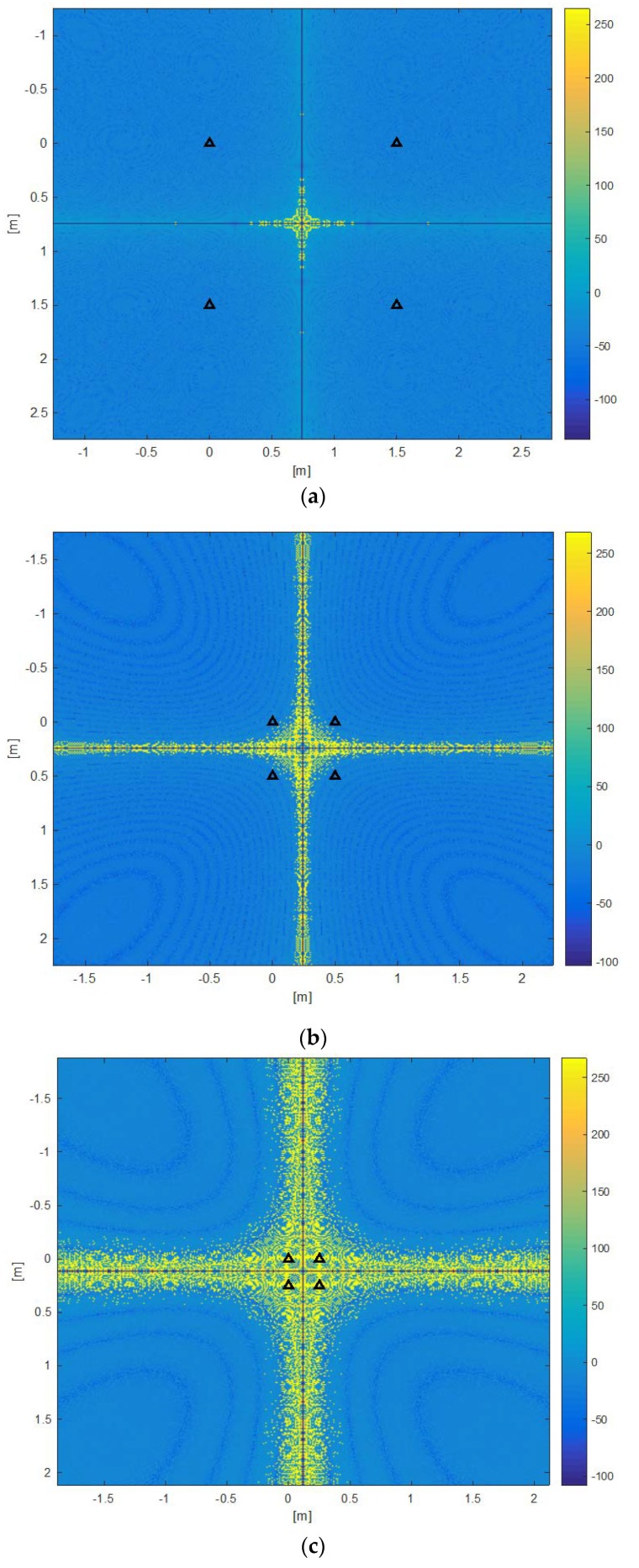
Estimation error of *l_1_* from (9) due to the quantization error on the estimates of *d_1_*, *d_2_*, and *d_3_* computed in a grid of points of z = 1.5 m, with different beacon set sizes: a) *a* = *b* = 1.5 m, b) *a* = *b* = 0.5 m, c) *a* = *b* = 0.25 m. Triangles show beacon positions. The error magnitude is in decibels (dB). Larger Beacon Sets considerably reduce the size of the “bad” region.

It is interesting to analyze by simulation the dependence of the error committed in the estimate of *l_1_* by (9) following the variation of *a* and *b*, which (9) does not explicitly show. Figure 15 shows the simulation estimation error trend of (9) due to the quantization error on the estimate of *d_1_*, *d_2_*, and *d_3_*, for a grid of points belonging to the plane *z* = 1.5 m (Beacon Set at z = 3), and by considering in sequence *a* = *b* = 1.5, 0.5, 0.25 m, respectively. Due to the very large dynamic of the error values, they are shown in decibels (dB).

As anticipated in Section 2, the accuracy of the current estimate of *l_1_*, and consequently of T_OFFSET_, from (9) strongly depends on the position of the MD with respect to the four beacons, but also on the beacon separation, as shown by Figure 15. The (9) produces bad estimates in some positions, where the denominator becomes very small or almost zero, as in the neighborhood of the “central cross” artifact visible in Figure 15. On the “central cross”, or in its neighborhood, due to the combined effect of the quantization error on the *d_i_s*, it results $d_{1}+d_{3}-d_{2}=0$ in many points with an unlimited error. In practice, the values of (9) are suitably limited with an adequate threshold tuned by trial and error procedure before feeding them to the ramp follower (10). Larger Beacon Sets considerably reduce the size of the “bad” region, as shown by Figure 15. On the other hand, smaller Beacon Sets are more easily integrated into room ceiling panels.

### 3.5. Discussion

Synchronization recovery between the reference set and MD required for accurate positioning is achieved using ultrasonic signals only, thus avoiding the costs, the board occupancy, and the power consumption of the RF section. This result is obtained by calculating the position through intersecting hyperboloids to recover synchronism, and then recalculating the same position using the acquired synchronism information, i.e., using intersecting spheres. In both cases intersections are computed by means of computationally efficient closed form formulas. In particular, the closed form formula for the calculation of the emitter-MD distance from the TDOAs is presented here for the first time.

Preliminary simulation using the MATLAB code has shown that the algorithm converges towards the estimation of the true T_OFFSET_ and that, after a certain transient time from the beginning of the operations, the synchronism is recovered, thus avoiding any RF reference signals. The clock drift problem afflicting every real system is solved with a suitable output estimator. The accuracy of the T_OFFSET_ estimate depends on the kind of estimator employed, in our case a ramp follower. As a drawback, while the ramp follower converges to the true value, it provides a sufficiently small error only after many estimation cycles. Estimation could be improved with a more refined estimator, which is however beyond the scope of this work.

The presented experimental setup provided both the reference positions and the positions estimated by the proposed technique for a point-to-point comparison. Comparing the two systems, the synchronized system [18] and the synchronization recovery-based system presented here, exactly the difference between the two systems due to the synchronization has been shown, while the rest of the hardware and the technique for measuring the TOA are identical. In this way, TOA and TDOA measurements underlie the same geometrical and noise conditions and localization results are comparable.

The experimental results obtained in a typical office room confirm the preliminary MATLAB code simulations, although obtained using a prototype with large margins for improvement. Positions with an error of less than 5 cm in respect to the reference system at a rate of 8 Hz were obtained.

In the proposed system architecture, each MD computes the positioning autonomously and privately, since it does not require any acknowledge message exchange similarly to GPS units, so that the same infrastructure can support positioning of an unlimited number of MDs. 

In fact, given a fixed infrastructure, composed of a set of ultrasonic emitters, any number of mobile devices can perform the positioning autonomously, requiring only a priori knowledge of the shape and periodicity of the signals emitted. Shape (e.g., chirp lower and higher frequencies) and periodicity in perspective can be also used to distinguish one emitter set from the neighbors in large spaces covered by multiple emitter sets in “cellular” way.

Mobile devices such as smartphones, tablets, and notebooks using appropriately low acoustic frequencies, can benefit, without hardware modifications, of the proposed positioning algorithm that can be simply executed as software by PCs or smartphones. In perspective, the use of commercial CMUT microphones such as the one used in our prototype would allow the use of higher frequencies, e.g., in the 30–50 kHz band, even on smartphones and PCs. Such a system paves the way to many applications and services, such as augmented reality, gestural interfaces, monitoring of the posture of the human body for recreational and medical purposes, training, indoor navigation of people, and devices within the framework of the Internet of Things.

## 4. Conclusions

In this work, a new system based on synchronization recovery for indoor mobile devices positioning was presented. The synchronization is obtained from TOA data using a closed form formula here presented for the first time. Recovered synchronization allows using spheres’ intersection instead of the hyperboloids’ intersection that is obtainable with the direct use of TDOA data. In this way, the proposed system achieves superior positioning accuracy. The presented experimental results are in good agreement with the simulation carried out for a typical office room, although obtained with a prototype with a large margin of improvement. Absolute positioning with an error of less than 5 cm in respect to the reference points and positioning rate of 8 Hz were obtained. Each MD computes the positioning autonomously and privately, and the same positioning infrastructure serves an unlimited number of MDs. Many applications and services within the Internet of Things can benefit of the presented system.
